# Monophyletic origin of domestic bactrian camel (*Camelus bactrianus*) and its evolutionary relationship with the extant wild camel (*Camelus bactrianus ferus*)

**DOI:** 10.1111/j.1365-2052.2008.01848.x

**Published:** 2009-08

**Authors:** R Ji, P Cui, F Ding, J Geng, H Gao, H Zhang, J Yu, S Hu, H Meng

**Affiliations:** * Key Laboratory of Dairy Biotechnology and Engineering Ministry of Education, College of Food Science and Engineering, Inner Mongolia Agricultural University010018 Huhhot, Inner Mongolia, China; † CAS Key Laboratory of Genome Sciences and Information, Beijing Institute of Genomics, Chinese Academy of Sciences100029 Beijing, China; ‡ Graduate School of the Chinese Academy of Sciences100029 Beijing, China; § School of Agriculture and Biology, Shanghai Jiao Tong University200240 Shanghai, China

**Keywords:** bactrian camel, domestication, mitochondrial genome, phylogeny

## Abstract

The evolutionary relationship between the domestic bactrian camel and the extant wild two-humped camel and the factual origin of the domestic bactrian camel remain elusive. We determined the sequence of mitochondrial *cytb* gene from 21 camel samples, including 18 domestic camels (three *Camelus bactrianus xinjiang*, three *Camelus bactrianus sunite*, three *Camelus bactrianus alashan*, three *Camelus bactrianus red*, three *Camelus bactrianus brown* and three *Camelus bactrianus normal*) and three wild camels (*Camelus bactrianus ferus*). Our phylogenetic analyses revealed that the extant wild two-humped camel may not share a common ancestor with the domestic bactrian camel and they are not the same subspecies at least in their maternal origins. Molecular clock analysis based on complete mitochondrial genome sequences indicated that the sub-speciation of the two lineages had begun in the early Pleistocene, about 0.7 million years ago. According to the archaeological dating of the earliest known two-humped camel domestication (5000–6000 years ago), we could conclude that the extant wild camel is a separate lineage but not the direct progenitor of the domestic bactrian camel. Further phylogenetic analysis suggested that the bactrian camel appeared monophyletic in evolutionary origin and that the domestic bactrian camel could originate from a single wild population. The data presented here show how conservation strategies should be implemented to protect the critically endangered wild camel, as it is the last extant form of the wild tribe Camelina.

## Introduction

The history and origin of the domestic camel remain elusive when compared with those of dog, donkey and pig (Giuffra *et al.* 2000; Savolainen *et al.* 2002; Beja-Pereira *et al.* 2004). Daniel Potts once said that if the Silk Road may be described as the bridge between the Eastern and Western cultures, the bactrian camel should rightfully be considered as the principal means of locomotion across that bridge (Potts 2005). The domestication of the bactrian camel, like many other domesticated mammals, has promoted unprecedented progress in cultural and economic development of human societies, representing a great leap forward for human civilization. Previous modern archaeological evidence suggests that the original habitat of the wild bactrian camel extended from the great bend of the Yellow River in north-western China through Mongolia to central Kazakhstan (Bannikov 1976; Schaller 1998; Nowak 1999), and it may have been domesticated in different regions of the East (multiple origins) about 5000 years ago, after which it subsequently spread westward towards Central Asia (Han *et al.* 2002). The domestic bactrian camel can be divided into six subspecies, *Camelus bactrianus xinjiang*, *Camelus bactrianus sunite*, *Camelus bactrianus alashan*, *Camelus bactrianus red*, *Camelus bactrianus brown* and *Camelus bactrianus normal*, according to the morphological characters. Bactrian camels are mainly herded in the cold desert areas of China and Mongolia and contribute significantly to the local economy (He 2002; Indra *et al.* 2003).

The extant wild bactrian camel, the only representative of the wild tribe Camelina as a result of the extinction of the wild dromedary, survives in north-western China and south-western Mongolia but is critically endangered (Hare 1997). Survival of the wild counterpart of the domestic bactrian camel (*Camelus bactrianus ferus*) in Mongolia and China has been long suspected, but convincing evidence has yet to be presented apart from the specimens described more than a century ago (Przewalski 1879). Despite the fact that *C. bactrianus ferus* has certain characters distinct from the domestic bactrian camel (*C. bactrianus*), such as the lower, pyramid-shaped humps, the thinner, lithe legs, and the smaller and more slender body, it is still difficult to tell them apart based on their morphological features alone. Therefore, it has been debated whether the extant wild camel is the progenitor of the domestic bactrian camel or whether the wild camel is actually an escapee from feral domestic camels, resulting from poor management of domestic herds (Zhao 1985). The fact that the identity and the genetic background of the wild two-humped camel remain obscure means that designing and implementing conservation programmes for the wild camel are difficult. Nevertheless, a previous molecular study focusing on restriction fragment length polymorphisms of the mitochondrial DNA (Han *et al.* 2002) demonstrated significant sequence variations between the wild camels and domestic bactrian camels from Alashan, Inner Mongolia and China, and proposed that the extant wild bactrian camel might be an independent species. Unfortunately, the limited sampling and poor resolution of the molecular markers provided little decisive information about the actual evolutionary relationship between them.

To understand better the evolutionary relationship between the extant wild camels and domestic bactrian camels, as well as the possible origin of the domestic bactrian camel, we determined the sequence of mitochondrial *cytb* genes from 21 camels, including 18 samples from domestic camels (three *C. bactrianus xinjiang*, three *C. bactrianus sunite*, three *C. bactrianus alashan*, three *C. bactrianus red*, three *C. bactrianus brown* and three *C. bactrianus normal*) and three samples from the wild *C. bactrianus ferus*. We compared the complete mitochondrial genomes from two wild and three domestic individual camels. In this report, we attempt to address the question about the evolutionary relationship of the two camels based on sequence variations.

## Materials and methods

### Specimens, DNA amplification and sequencing

Ear samples of the bactrian camel were collected from several areas across the cold desert region of China and Mongolia in 2006. The sampling locations are listed in Table S1. Genomic DNA was extracted according to proteinase K/phenol extraction method. A PCR-based approach for mitochondrial genome sequencing was used (Yamauchi *et al.* 2004). The PCR primers (Table S2) used for the initial amplification were designed based on the mitochondrial genome sequence of wild camel (*C. bactrianus ferus*) from public databases. The raw sequence data were acquired to achieve at least threefold coverage of the entire genome in order to assure sequence quality and accuracy. The PCR primers used for amplification of the mitochondrial *cytb* gene are also listed in Table S2.

Standard PCRs were conducted in a 25-μl reaction volume containing 1 or 2 U *Taq* DNA polymerase, 10 mm Tris–HCl (pH 8.3), 0.25 mm dNTPs, 0.2–2 mm bovine serum albumin (BSA), 1.5–2.5 mm MgCl_2_, 20 pm of each primer and about 10 ng camel genomic DNA. The PCR reaction conditions were set as: 94 °C for the first 5 min, followed by 35 cycles of 94 °C denaturation for 30 s, 50 °C annealing for 30 s and 72 °C extension for 45 s.

The thermo-cycling sequencing reaction was performed in a final volume of 24 μl containing 8 μl DYEnamic ET Terminator Sequencing Kit premix, 10 pm sequencing primers and 50 ng DNA. The reactions were carried out at 95 °C for 2 min, followed by 35 cycles of 95 °C denaturation for 15 s, 50 °C annealing for 15 s and 60 °C extension for 90 s. The amplified DNA fragments were sequenced with an ABI-3730 sequencer. The primers for PCR reactions were also used for the sequencing reaction from each direction. DNA sequences were assembled by using the software package phred/phrap/consed/ (Ewing & Green 1998; Gordon *et al.* 1998) on a PC/UNIX platform. The mitochondrial sequences were annotated with BLAST tools, and tRNA genes and their secondary structures were identified according to trnascan-se (Lowe & Eddy 1997).

### Phylogenetic analysis

The phylogenetic trees were reconstructed according to a neighbour-joining method implemented in mega (Kumar *et al.* 2004) and the maximum likelihood method implemented in phylip (Felsenstein 1989). The reliability of the branches was assessed by bootstrap analysis (1000 bootstrap replications). Bayesian posterior probability of phylogeny was performed with mrbayes (MCMC method with 1 000 000 generations) (Ronquist & Huelsenbeck 2003). Two different models were used: the General Time Reversible and the Hasegawa, Kishino and Yano (HKY) models. Nucleotide divergence was estimated using Kimura’s (1980) two-parameter method implemented in mega. The molecular clock was tested with Tajima’s (1993) test; when the wild camel and domestic bactrian camel were tested, dromedary was used as an outgroup.

## Results

### Phylogeny of the domestic bactrian camel and its extant wild counterpart

**Figure 1:**
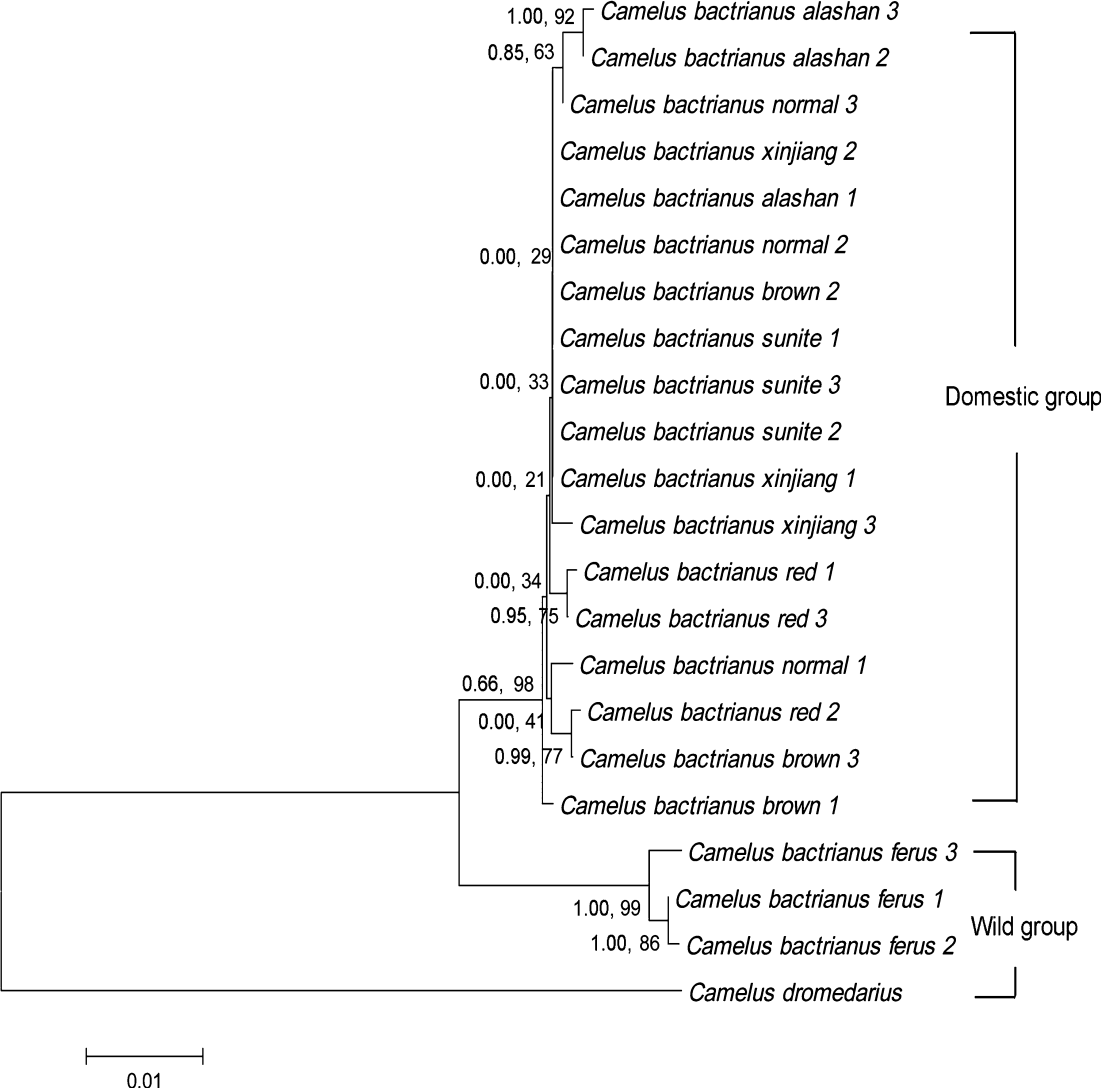
Majority-rule (20%) neighbour-joining tree of *cytb* gene sequences inferring the phylogeny of bactrian camels. The topology is rooted with *Camelus dromedarius* as the outgroup, based on the maximum likelihood method. The nodes were supported in the boostrap value (1000 replications) and posterior probability shown on each node (the number on the right is the bootstrap value and the number on the left is the posterior probability). The scale bar indicates 1% sequence divergence.

Based on the *cytb* sequences from the samples (Table S1), we constructed phylogenetic trees and used the corresponding *C. dromedarius* sequence (the closest extant taxon) as the outgroup (Fig. 1). Similar topology was observed based on both neighbour-joining and maximum likelihood methods. The results showed two highly divergent phylogenetic clades with an average genetic distance of 2.8 ± 0.5% (the sequence divergence is 26–33 substitutions) between the domestic and wild camels, supported by high bootstrap values and Bayesian posterior probability, which suggests that the domestic and wild camels may belong to two different lineages. In addition, the lower bootstrap values in the domestic clade suggested a relatively low sequence divergence (0.2 ± 0.1%) among the *cytb* sequences from the individual domestic camel.

### Evolutionary relationships between the extant wild camels and the domestic bactrian camel

To further investigate the evolutionary relationship of the wild camel and the domestic bactrian camel, we sequenced two mitochondrial genomes from the wild camels (*C. bactrianus ferus*) and three domestic camels (*C. bactrianus alashan*). We first examined the intersubspecific variations that are important in understanding the evolutionary history of the bactrian camel. We compared five complete mitochondrial genome sequences, excluding intrasubspecific variations, and identified 195 substitutions, including 178 transitions and 17 transversions. The rate of transitions was much higher than that of transversions, in common with other vertebrate mitochondrial genomes. The number of substitutions in the protein-coding sequence is higher (168 substitutions) compared with the control regions (15 substitutions) and RNA genes (12 substitutions). We also found 22 non-synonymous substitutions among *nd2*, *nd3*, *nd4*, *nd5*, *nd6* and *cytb* genes (Table 1) and 17 amino acid variations between hydrophobic and hydrophilic proteins, implying possible functional alterations.

**Table 1:** Intersubspecific non-synonymous substitutions in protein-coding regions between the wild and domestic camels.

Gene	Codon variation in wild/domestic (W/D)	Amino acid variation in W/D	Position in gene
*nd2*	CTT/ATT; GAC/GGC	L/I; D/G	727; 956
*nd3*	ACC/ATC	T/I	260
*nd4*	AAC/ATC; TCT/GCT; TCC/ACC	N/I; S/A; S/T	1431; 781; 303
*nd5*	GCA/ACA; ATA/CTA; CTC/CAC	A/T; M/L; L/H	484; 1486; 1538
	ATT/GTT; ACA/ATA; GTG/ATG	I/V; T/M; V/M	1609; 1631; 1651
	ATT/GTT; ATC/ACC	I/V; I/T	1777; 1787
*nd6*	ATT/GTT; TCT/GCT; ATT/GTT	V/I; A/S; V/I	34; 304; 466
*cytb*	ACC/ATC; CAT/CGT; ATC/GTC	T/I; V/M; I/V	12; 51; 115
	GTA/ATA; GTA/ATA; GCA/GTG	V/M; V/M; A/V	706; 1045; 1058

**Figure 2:**
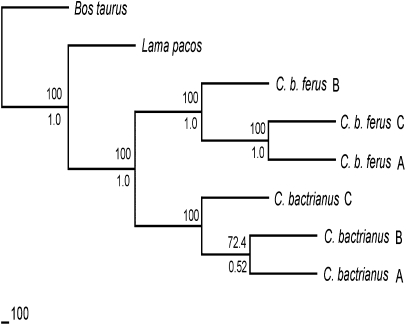
The construction of the phylogenetic relationship between *Camelus bactrianus* and *C. bactrianus ferus* based on the complete mitochondrial genomic sequences, excluding the control regions. The tree was reconstructed based on the maximum likelihood method. To estimate the divergence time, alpaca (*Lama pacos*) was taken as an in-group calibration point; all nodes were supported by the bootstrap value (1000 replications) and posterior probability shown on each node (bootstrap value is above the branch and posterior probability is below the branch). The scale bar indicates 100 substitutions.

Based on these variations, we constructed a phylogenetic tree based on the five newly acquired complete mitochondrial genome sequences and the available sequence data from the wild camel (*C. bactrianus ferus*), using alpaca and cow as outgroups (Fig. 2). As the control region of mtDNA has a high incidence of homoplasy (Ingman *et al.* 2000; Bjornerfeldt *et al.* 2006), we excluded it from this analysis; we observed similar topology with both neighbour-joining and maximum likelihood methods.

Based on the phylogeny and the sequence data, we dated the divergence time between the wild and domestic camels. As the estimation can be carried out with or without assumption of a constant evolutionary rate among all compared clades, we used the likelihood ratio method to perform the evolutionary rate constancy test (see Materials and methods). The result showed significant differences in the evolutionary rate between the two subspecies (without rate constancy Ln *L*_1_ = −39805.676850; with rate constancy Ln *L*_2_ = −39854.056320; *P* < 0.01) and rejected the assumption of a constant rate among the camel mitochondrial genomes.

The study demonstrated that the assumption of rate constancy might be inappropriate for estimating divergence time between the wild and domestic camels. Therefore, we used a heuristic rate-smoothing procedure for ML-based estimates (Yang 2004), which takes into account the evolutionary rates among different branches of the tree. We used one fossil calibration point: 11 million years, the estimated time for divergence of camel and alpaca (Webb 1974; Stanley *et al.* 1994). Therefore, the divergence time between the wild and domestic camels was estimated to be 0.7 million years (early Pleistocene). According to the archaeological dates of the earliest known bactrian camel domestication (5000–6000 years ago) (Han *et al.* 2002), we can conclude that the extant wild camel is a separate lineage to, but not the direct progenitor of, the domestic bactrian camel.

### Haplotypes of *cytb* genes from 21 individuals

Among the *cytb* sequences, we identified 14 haplotypes that are divided into two haplogroups: the domestic (D) haplogroup and the wild (W) haplogroup (Fig. S1). The domestic haplogroup includes 11 haplotypes, of which haplotype D1 shows the highest frequency in domestic camels (eight individuals). Other haplotypes are present in fewer individuals (Table S3). Among the wild haplogroups, there are three haplotypes; each is contributed by a single wild individual.

## Discussion

To further investigate the evolutionary relationship between the extant wild camels and domestic bactrian camels, we sequenced five mitochondrial genomes from two wild and three domestic individuals, using 23 pairs of universal PCR primers for the initial amplification based on the mitochondrial sequence of *C. bactrianus ferus* from the public databases (Table S2 and Fig. S2). The three mitochondrial genome sequences from *C. bactrianus* are 16 659–16 667 bp in length, shorter than that of *C. bactrianus feru*s (Cui *et al.* 2007). Minor length variations occurred in the tandem repeat (ACGTAC)_*n*_ of the control region. The gene order and content of the bactrian mitochondrial genome are similar to those of other placental mammals (Fig. S2); it harbours 13 protein-coding genes (three subunits of the *cytochrome c oxidase* gene, genes coding for seven subunits of the NADH ubiquinone oxidoreductase complex, one gene coding for one subunit of the ubiquinol cytochrome b oxidoreductase complex and genes coding for two subunits of ATP synthases), the small and large ribosomal RNA genes, and 22 tRNA genes. The replication origin of the light strand within a tRNA gene cluster was also unambiguously identified (Table S4). The mitochondrial sequence from two wild camels is highly homologous to a previously reported sequence of *C. bactrianus feru*s (Cui *et al.* 2007).

In this study, we reconstructed the phylogeny of the bactrian camels, proposing that the extant wild bactrian camel and domestic bactrian camel have separate maternal origins and that the two subspecies diverged some 0.7 million years ago. Archaeological evidence suggests that the bactrian camel migrated from North America to Asia via the Bering Strait near the end of the Tertiary period, some 3 million years ago (Harrison 1985). In the Pleistocene Epoch (the Great Ice Age), about 1.8 million–10 000 years ago, possibly because of vast climate changes, a large-scale migration of the bactrian camel population may have occurred again, leading to the split of the two lineages. However, at present, there is little fossil evidence supporting the hypothesis of the second large-scale migration.

We consider that the extant wild camel belongs to a distinct subspecific lineage that is different from the progenitor of its domesticated counterpart; both evolved from the North America populations. At present, the wild camel is critically endangered and only several hundred wild camels have survived in their original habitats, a region covering the north-western China and south-western Mongolia. The available sequence data, such as the five highly similar *cytb* gene sequences from the wild camels found in Gansu, China, and Govi-Altay, Mongolia, showed that the wild camels surviving in China and Mongolia belong to the same lineage. We have failed to identify the direct descendant of the extant wild camel despite having sampled domestic individuals extensively. Therefore, we believe that the extant wild camels may have originated from the escapees of the domesticated counterparts. We would like to campaign for improved conservation guidelines for these wild camels.

As there are very limited morphological differences between the extant wild and domestic bactrian camels, the relationship between the groups was proposed as subspecific (Reading *et al.* 2002). However, recent behavioural observations have shown that their interbreeding descendants almost lose the ability to reproduce, suggesting that they should be classified as different species. To address the controversy, we analysed the 13 mitochondrial protein-coding genes over the mammalian lineage. We classified these sequences available from the public databases into different hierarchical (taxonomical) structures and estimated the average divergences for subspecies, species, genus, family and order, and obtained the corresponding divergence values of 3.8 ± 1.4%, 7.6 ± 0.75%, 15.9 ± 0.94%, 27.8 ± 0.94%, and 35.1 ± 0.45% (data not shown) respectively. The sequence divergence between the wild and domestic camels was estimated as 2.4 ± 0.2%, that is, less than the average divergence between species within a genus, but falling into the range for subspecies. Therefore, both morphological and molecular evidence suggests that the wild and domestic camels are subspecific rather than specific, despite the fact that some between-species divergence values are actually lower than the average.

The bactrian camel was mainly domesticated in China and Mongolia, but there have not been population studies reported thus far. Based on the phylogenetic tree, we observed lower bootstrap values in the domestic branches (Fig. 1) and we believe that the six subspecies were domesticated from a single ancestral wild population, i.e. the domestic bactrian camel is monophyletic in origin. The direct progenitor of the domestic bactrian camel may have gone extinct long before humans began to pay attention to these useful animals. According to the distribution of the domestic bactrian camels and modern archaeological evidence, the original habitat of the ancestor of the domestic bactrian camel extended from the great bend of the Yellow River in the Gansu Province of north-west China through Mongolia to Central Kazakhstan. We believe that the bactrian camel was first domesticated in the cold desert region of China and Mongolia. However, we cannot exclude the possibility that domestication of bactrian camel may have occurred in regions other than those studied here.

### Accession numbers

The GenBank (http://www.ncbi.nlm.nih.gov) accession numbers for the complete mitochondrial genome sequences determined in this study are *C. bactrianus ferus* (EF507800, EF507801), *C. bactrianus* (EF212037, EF507798, EF507799). The accession numbers of *cytb* determined in this study are listed in Table S1. The GenBank accession numbers for the complete mitochondrial genome sequences of the individual *C. bactrianus ferus*, *Lama pacos* and *Bos taurus* are EF212038, Y19184 and AY526085 respectively. The GenBank accession number for the *C. dromedarius cytb* gene is U06426, and the GenBank accession numbers for the *C. bactrianus ferus cytb* genes are AY126622.1, AY126618.1, EF076243.1, AY126624.1 and EF076246.1.

## Supporting information

**Figure S1** The haplotypes of *cytb* genes among 21 camels.

**Figure S2** The mitochondrial genome of *Camelus bactrianus*.

**Table S1** Bactrian camel mitochondrial genome sequences used for reconstructing the phylogenetic tree.

**Table S2** PCR primers used.

**Table S3** Distribution of *cytb* haplotypes among the 18 domestic and three wild camels.

**Table S4** Organization of the *Camelus bactrianus* mitochondrial genome.
