# Supplementary material for: Monophyletic origin of domestic bactrian camel (Camelus bactrianus) and its evolutionary relationship with the extant wild camel (Camelus bactrianus ferus)
Source: Anim Genet. 2009 Aug;40(4):377–82. doi: 10.1111/j.1365-2052.2008.01848.x (PMC2721964; doi:10.1111/j.1365-2052.2008.01848.x)
Supplement: Supplementary file 1 [file age0040-0377-SD1.pdf]

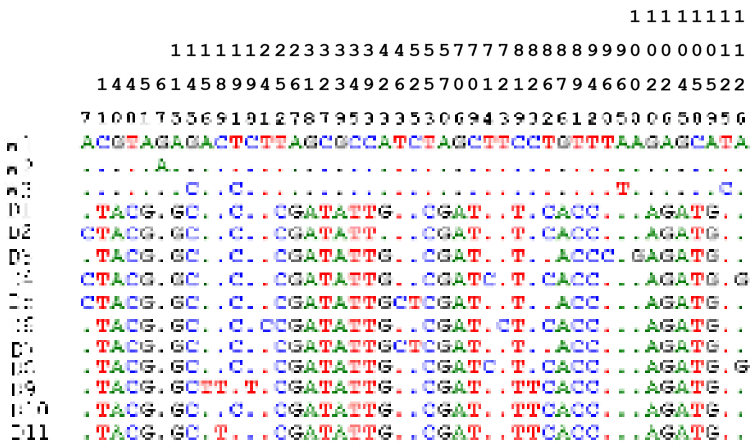

Figure S1. The haplotypes of *cytb* genes among 21 camels. W1, W2, and W3 indicate three haplotypes identified in three wild individuals; D1 to D11 indicate 11 haplotypes found in 18 domestic camel individuals. The numbers on top indicate the position of single nucleotide variations. The points in the figure indicate the same base as the reference (W1).
