# Supplementary material for: Monophyletic origin of domestic bactrian camel (Camelus bactrianus) and its evolutionary relationship with the extant wild camel (Camelus bactrianus ferus)
Source: Anim Genet. 2009 Aug;40(4):377–82. doi: 10.1111/j.1365-2052.2008.01848.x (PMC2721964; doi:10.1111/j.1365-2052.2008.01848.x)
Supplement: Supplementary file 2 [file age0040-0377-SD2.pdf]

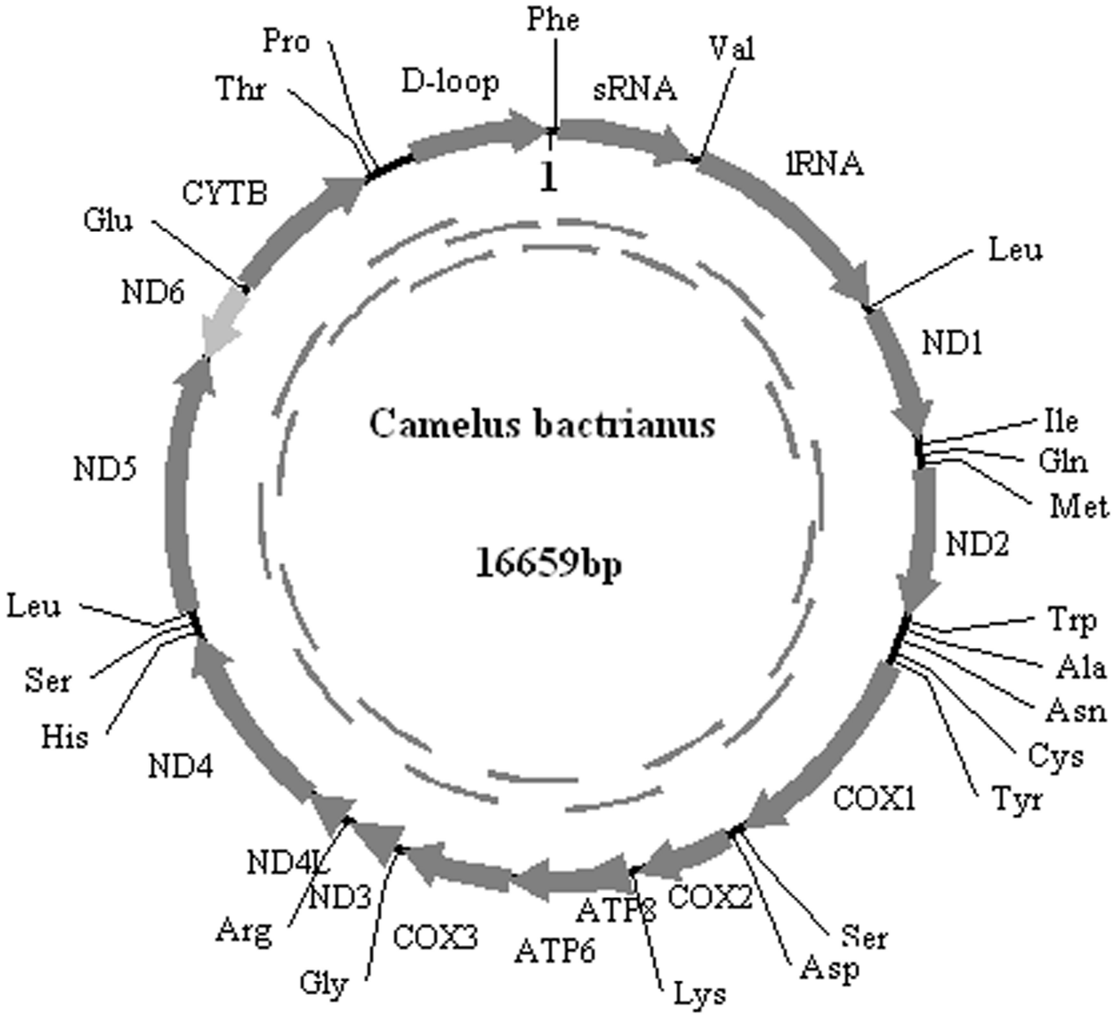

Figure S2. The mitochondrial genome of *C. bactrianus*. Protein-coding genes (black) and rRNA genes are denoted by arrows. The *nd6* gene (light gray) is transcribed in the opposite direction relative to the rest in the cluster. tRNA genes are depicted with their corresponding amino acids. The overlapped PCR fragments covering the entire mitochondrial genome are also indicated.
