# Supplementary material for: Monophyletic origin of domestic bactrian camel (Camelus bactrianus) and its evolutionary relationship with the extant wild camel (Camelus bactrianus ferus)
Source: Anim Genet. 2009 Aug;40(4):377–82. doi: 10.1111/j.1365-2052.2008.01848.x (PMC2721964; doi:10.1111/j.1365-2052.2008.01848.x)
Supplement: Supplementary file 3 [file age0040-0377-SD3.pdf]

Table S1. Bactrian camel mitochondrial genome sequences used for reconstructing the phylogenic tree

Species	No.of samples	Origin	Accession number
Camelus bactrianus xinjiang	3	BoLe, XinJiang, China	FJ171705, FJ171708, FJ171713
Camelus bactrianus sunite	3	ShuNite, Inner Mongolia, China	FJ171699, FJ171706, FJ171711
Camelus bactrianus alashan	3	ALashan, Inner Mongolia, China	FJ171702, FJ171714, FJ171716
Camelus bactrianus red	3	DungGovi, Mongolia	FJ171703, FJ171704, FJ171712
Camelus bactrianus brown	3	ÖmnöGovi, Mongolia	FJ171700, FJ171707, FJ171710
Camelus bactrianus normal	3	Govi-Altay, Mongolia	FJ171701, FJ171709, FJ171715
Camelus bactrianus ferus	3	Govi-Altay, Mongolia	EF212038, EF507800, EF507801
