# Supplementary material for: Monophyletic origin of domestic bactrian camel (Camelus bactrianus) and its evolutionary relationship with the extant wild camel (Camelus bactrianus ferus)
Source: Anim Genet. 2009 Aug;40(4):377–82. doi: 10.1111/j.1365-2052.2008.01848.x (PMC2721964; doi:10.1111/j.1365-2052.2008.01848.x)
Supplement: Supplementary file 4 [file age0040-0377-SD4.pdf]

Table S2. PCR primers used

Primer	Sequence(5′-3′)	5′Position	Strand
lt1	ATCCCAGCCTTTCTATTAG	78	upper
lt2	CCTAGTTCAATCAAGCACTC	898	lower
lt3	AAAACGTTAGGTCAAGGTG	740	upper
lt4	CTCTCTAAGGAAGGTTGTATC	1562	lower
lt5	TGTCCAGGAAATGAATATAAG	1456	upper
lt6	AATTGCTAACTCAGGTAGAAG	2240	lower
lt7	TAAGACGAGAAGACCCTATG	2145	upper
lt8	CGATGATGAACATAGTAACAG	2962	lower
lt9	CCTAGGCTACATGCAACTC	2823	upper
lt10	ACACGTGTCATATGCATAAG	3658	lower
lt11	AACCGCAACTTTCCTATG	3540	upper
lt12	AGCCTCCAACTATAATTGAC	4406	lower
lt13	TCTCCGTACTGTACCAAATC	4323	upper
lt14	TTGAAGCCAGCTGACTAG	5161	lower
lt15	GCTAAGCCCTTACTAGATTG	5081	upper
lt16	AGAGAACGGCAGTGATTAG	5916	lower
lt17	ATTTAGCGGGAGTATCTTC	5785	upper
lt18	GTAGTCAGAGTAGCGTCGAG	6662	lower
lt19	CACGATTGACGATACATGAG	6542	upper
lt20	TGGTTTTAGGTCTGATGTTG	7411	lower

lt21	AGCTACGAGTATACGGACTAC	7340	upper
lt22	AGTCCTAGGAGATTGGTAGTC	8197	lower
lt23	TAATCCGACTCACATCTAAAC	8083	upper
lt24	AGTGATAAAAGGCTCAGAAG	8927	lower
lt25	AGACGTTGTCCGAGAAAG	8811	upper
lt26	AGGTCAAATAGGAGGAATG	9671	lower
lt27	CCCAACTATACATTTATGCAG	9545	upper
lt28	GGCAGTAGTCATGTCGTTAG	10397	lower
lt29	CTCCACGCTTAATCAGTATAG	10313	upper
lt30	AACTATAAGGGTCGTAGCTC	11127	lower
lt31	CACTTATTGCATATTCCTCAG	11047	upper
lt32	TCTTGGTTCGAATGGATG	11932	lower
lt33	CAACACCTACAACAACAATC	11825	upper
lt34	CCAACTGGCTTGAAGTAG	12684	lower
lt35	AACCTTAACCCTATGCTTG	12593	upper
lt36	CTCCAGTCAAAGTAGGTCTAG	13430	lower
lt37	CTTCTTTCCCACTATTATACAC	13334	upper
lt38	AAGCAGATTCCTAGTAGAGAG	14281	lower
lt39	AACACCCGAAAATCACAC	14183	upper
lt40	TGCTAGGATTAGGATAGAGAG	15065	lower
lt41	CCTCAATACACCACCACAC	14960	upper
lt42	AGATGGTCCTGAAGTAAGAAC	15811	lower

lt43	TTAGATCACGAGCTTAATCAC	15668	upper
lt44	TGTGGCTACTTGAGAGATTG	16547	lower
lt45	CGCATTCAGCAAGTATTTAG	16380	upper
lt46	ACCTTAGCTGTCGTGTAGTAG	482	lower
Cytb1	GTACGTCATCATATATTCC	14074	upper
Cytb2	TAGGTGTACGGCTACTAG	15141	lower
Cytb3	TTATTCGATACCTACATGC	14392	upper
Cytb4	GATAAGTGCTAGGATTAGG	15053	lower
Cytb5	TGCCATTTATTATCACAGC	14713	upper
Cytb6	TCTTCCCTGAGTCTTAGG	15360	lower
