# Supplementary material for: Monophyletic origin of domestic bactrian camel (Camelus bactrianus) and its evolutionary relationship with the extant wild camel (Camelus bactrianus ferus)
Source: Anim Genet. 2009 Aug;40(4):377–82. doi: 10.1111/j.1365-2052.2008.01848.x (PMC2721964; doi:10.1111/j.1365-2052.2008.01848.x)
Supplement: Supplementary file 5 [file age0040-0377-SD5.pdf]

Table S3. Distribution of *cytb* haplotypes among the 18 domestic and three wild camels

**Haplogroups**	**Haplotypes**	*C.bactrianus xinjiang*	*C.bactrianus sunite*	*C.bactrianus alashan*	*C.bactrianus red*	*C.bactrianus brown*	*C.bactrianus normal*	*C.bactrianus ferus*
Domestic(D)	D1	2	3	1	0	1	1	0
	D2	0	0	0	0	1	0	0
	D3	0	0	0	0	0	1	0
	D4	0	0	0	1	0	0	0
	D5	0	0	0	1	0	0	0
	D6	1	0	0	0	0	0	0
	D7	0	0	0	0	1	0	0
	D8	0	0	0	1	0	0	0
	D9	0	0	1	0	0	0	0
	D10	0	0	0	0	0	1	0
	D11	0	0	1	0	0	0	0
Wild (W)	W1	0	0	0	0	0	0	1
	W2	0	0	0	0	0	0	1
	W3	0	0	0	0	0	0	1
