# Supplementary material for: Monophyletic origin of domestic bactrian camel (Camelus bactrianus) and its evolutionary relationship with the extant wild camel (Camelus bactrianus ferus)
Source: Anim Genet. 2009 Aug;40(4):377–82. doi: 10.1111/j.1365-2052.2008.01848.x (PMC2721964; doi:10.1111/j.1365-2052.2008.01848.x)
Supplement: Supplementary file 6 [file age0040-0377-SD6.pdf]

Table S4. Organization of the *Camelus bactrianus* mitochondrial genome

Gene	Start	End	Strand	Length
Phe tRNA	1	67	+	67
s-rRNA	68	1035	+	968
Val tRNA	1035	1100	+	66
l-rRNA	1101	2665	+	1565
Leu tRNA	2666	2740	+	75
NADH dehydrogenase subunit 1	2744	3699	+	318
Ile tRNA	3700	3768	+	69
Gln tRNA	3766	3838	-	73
Met tRNA	3840	3908	+	69
NADH dehydrogenase subunit 2	3909	4950	+	347
Trp tRNA	4951	5018	+	68
Ala tRNA	5024	5092	-	69
Asn tRNA	5094	5166	-	73
Cys tRNA	5200	5266	-	67
Tyr tRNA	5267	5333	-	67
cytochrome c oxidase subunit I	5335	6879	+	514
Ser tRNA	6881	6951	-	71
Asp tRNA	6955	7021	+	67
cytochrome c oxidase subunit II	7023	7706	+	227
Lys tRNA	7710	7776	+	67

ATP synthase F0 subunit 8	7778	7981	+	67
ATP synthase F0 subunit 6	7939	8619	+	226
cytochrome c oxidase subunit III	8619	9402	+	261
Gly tRNA	9403	9472	+	70
NADH dehydrogenase subunit 3	9470	9818	+	116
Arg tRNA	9820	9887	+	68
NADH dehydrogenase subunit 4L	9888	10184	+	98
NADH dehydrogenase subunit 4	10178	11555	+	459
His tRNA	11555	11624	+	70
Ser tRNA	11625	11683	+	59
Leu tRNA	11685	11754	+	70
NADH dehydrogenase subunit 5	11755	13575	+	606
NADH dehydrogenase subunit 6	13559	14086	-	175
Glu tRNA	14088	14156	-	69
cytochrome b	14161	15300	+	379
Thr tRNA	15301	15369	+	69
Pro tRNA	15369	15434	-	66
